# Organ Specificity and Commonality of Epigenetic Aging in Low‐ and High‐Running Capacity Rats

**DOI:** 10.1111/acel.70110

**Published:** 2025-06-08

**Authors:** Takuji Kawamura, Csaba Kerepesi, Juliet Polok Sarkar, Ferenc Torma, Zoltan Bori, Lei Zhou, Peter Bakonyi, Attila Kolonics, Laszlo Balogh, Mitsuru Higuchi, Vivien Pillár, Karolina Pircs, Lauren Gerard Koch, Steven Loyal Britton, Erika Koltai, Zsolt Radak

**Affiliations:** ^1^ Research Center for Molecular Exercise Science Hungarian University of Sports Science Budapest Hungary; ^2^ Smart‐Aging Research Center Tohoku University Sendai Japan; ^3^ Institute for Computer Science and Control (SZTAKI) Budapest Hungary; ^4^ Hun‐Ren‐SZTAKI‐SU Rejuvenation Research Group, Office for Supported Research Groups (TKI), Hungarian Research Network (HUN‐REN) Budapest Hungary; ^5^ Doctoral School of Informatics, Faculty of Informatics Eötvös Loránd University Budapest Hungary; ^6^ Laboratory of Exercise Biochemistry and Neuroendocrinology, Faculty of Health and Sport Science University of Tsukuba Tsukuba Ibaraki Japan; ^7^ Institute of Sport Sciences University of Debrecen Debrecen Hungary; ^8^ Faculty of Sport Sciences Waseda University Saitama Japan; ^9^ Institute of Translational Medicine Semmelweis University Budapest Hungary; ^10^ Hungarian Centre of Excellence for Molecular Medicine ‐ Semmelweis University (HCEMM‐SU), Neurobiology and Neurodegenerative Diseases Research Group Budapest Hungary; ^11^ Laboratory of Molecular Neurogenetics, Department of Experimental Medical Science, Wallenberg Neuroscience Center and Lund Stem Cell Center Lund University Lund Sweden; ^12^ Department of Physiology and Pharmacology The University of Toledo College of Medicine and Life Sciences Toledo Ohio USA; ^13^ Department of Anesthesiology University of Michigan Ann Arbor Michigan USA; ^14^ Institute of Sport Sciences and Physical Education, Faculty of Sciences University of Pécs Pécs Hungary; ^15^ Department of Bioengineering Sapientia Hungarian University of Transylvania Miercurea Ciuc Romania

**Keywords:** epigenetic drift, global DNA methylation, intersection clock, maximal oxygen uptake, methylation entropy, rat epigenetic clock

## Abstract

Epigenetic drift, which is gradual age‐related changes in DNA methylation patterns, plays a significant role in aging and age‐related diseases. However, the relationship between exercise, epigenetics, and aging, and the molecular mechanisms underlying their interactions are poorly understood. Here, we investigated the relationship between cardiorespiratory fitness (CRF), epigenetic aging, and promoter methylation of individual genes across multiple organs in selectively bred low‐ and high‐capacity runner (LCR and HCR) aged rats. Epigenetic clocks, trained on available rat blood‐derived reduced representation bisulfite sequencing data, did not reflect differences in CRF between LCR and HCR rats across all four organs. However, we observed organ‐specific differences in global mean DNA methylation and mean methylation entropy between LCR and HCR rats, and the direction of these differences was the opposite compared to the age‐related changes in the rat blood. Notably, the soleus muscle exhibited the most pronounced differences in promoter methylation due to CRF. We also identified seven genes whose promoter methylation was consistently influenced by CRF in all four organs. Moreover, we found that age acceleration of the soleus muscle was significantly higher compared to the heart and the hippocampus, and significantly lower compared to the large intestine. Finally, we found that the age acceleration was not consistent across organs. Our data suggest that CRF associates with epigenetic aging in an organ‐specific and organ‐common manner. Our findings provide important insights into the biology of aging and emphasize the need to validate rejuvenation strategies in the context of the organ‐specific nature of epigenetic aging.

## Graphical abstract

Cardiorespiratory fitness (CRF) is not associated with epigenetic clocks, including newly developed ones. On the other hand, CRF is associated with global methylation profiles and promoter methylation in organ‐specific and organ‐common manner.

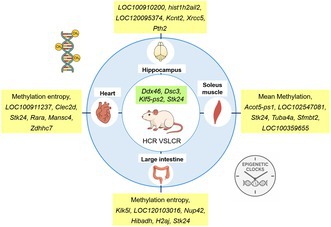

**Abbreviations**
CRF: cardiorespiratory fitness
GMM: global mean DNA methylation
HCR: high‐capacity runner
LCR: low‐capacity runner
MAE: mean absolute error
MedAE: median absolute error
MME: mean methylation entropy
rDNA: ribosomal DNA
RRBS: reduced representation of bisulfite sequencing
VO_2max_
: maximal oxygen uptake

## 1 Introduction

Aging is a complex biological process characterized by a gradual decline in physiological integrity, which leads to impaired function and increased vulnerability to death (López‐Otín et al. 2013). The aging rate is influenced by various genetic and environmental factors. Among these factors, interest in epigenetics as an important regulator of aging has been increasing. Epigenetic modifications, including DNA methylation, histone modifications, and non‐coding RNA regulation, play a pivotal role in regulating gene expression patterns and consequently contribute significantly to the aging process. Notably, alterations in DNA methylation patterns during aging, termed epigenetic drift, occur across various tissues and cells and contribute to the progressive dysregulation of gene expression. Therefore, clinical trials have begun to evaluate the efficacy of rejuvenation strategies targeting epigenetic drift, such as lifestyle interventions, plasmapheresis, and the utilization of drugs and dietary supplements (Moqri et al. 2023).

The beneficial effects of exercise on healthy aging are well documented. In particular, cardiorespiratory fitness (CRF), represented by maximal oxygen uptake ($\dot{V}$O_2max_), is an indicator of longevity (Blair et al. 1989; Wei et al. 1999). However, understanding the relationship among exercise, epigenetics, and aging is a complex endeavor, and the molecular mechanisms underlying this interaction remain an ongoing scientific pursuit. Previous research has demonstrated the influence of acute and regular exercise on global and gene‐specific promoter methylation, primarily in peripheral blood and skeletal muscle (Barrès et al. 2012; Voisin et al. 2024). Recent findings, including those of our study, also indicate a negative relationship between physical fitness (including activity levels and CRF) and epigenetic aging based on age‐related changes in DNA methylation levels at CpG sites (Fox et al. 2023; Jokai et al. 2023; Kawamura et al. 2024; Quach et al. 2017). These lines of evidence suggest that exercise exerts a rejuvenating effect on epigenetic aging and has a favorable effect on the aging process. Importantly, the health‐promoting effects of maintaining and improving CRF extend to multiple organs and the whole body (Ashcroft et al. 2024). However, only a conference abstract reported the relationship between CRF and epigenetic aging across multiple organs (Rossiter et al. 2023). To the best of authors’ knowledge, no study has explored the organ specificity and commonality of promoter methylation in individual genes according to differences in CRF.

Based on extensive epidemiological studies that have shown that CRF is a strong predictor of morbidity and mortality (Blair et al. 1989; Wei et al. 1999), Koch and Britton formulated the “aerobic hypothesis” that variation in the capacity for oxygen metabolism is the central mechanistic determinant of the difference between complex diseases and health (Koch et al. 2012). To test this hypothesis, low‐ and high‐running capacity strains were established by divergent selection breeding for running capacity using a founder population of a genetically heterogeneous N:NIH rat stock (i.e., eight inbred lines: ACI, BN, BUF, F344, M520, MR, WKY, WN) (Koch and Britton 2001; Koch et al. 2011). Notably, CRF, measured throughout adulthood, was a reliable predictor of lifespan, and the median lifespan was extended by 8–10 months in high‐capacity runners (HCRs) than in low‐capacity runners (LCRs) without extending maximum lifespan (Koch et al. 2011). This finding implies that the LCR rats were more susceptible to disease, supporting the healthspan‐extending effects (not lifespan‐extending effects) of exercise. Therefore, this rat strain is ideal for elucidating the unknown mechanisms underlying the correlation between CRF and healthspan, and how CRF regulates epigenetic aging and promoter methylation, which are associated with morbidity and mortality, across multiple organs.

In this study, we performed reduced representation bisulfite sequencing (RRBS) on the hippocampus, heart, soleus muscle, and large intestine of selectively bred LCR and HCR aged rats, which are known for their different disease risks and life expectancy (Koch et al. 2011; Wisløff et al. 2005). Our aim was to elucidate the relationship between CRF, epigenetic aging, and promoter methylation of individual genes across multiple organs in this rat model.

## 2 Methods

### 2.1 Experimental Animals

Sixteen female LCR and HCR rats of the 44th generation (23–24 months old) were used in this study. LCR and HCR rat models were obtained by artificially selecting low‐ and high‐running capacities from genetically heterogeneous N:NIH stock rats (Koch and Britton 2001). Rats were transported by air from The University of Toledo (Toledo, OH, USA) to the Hungarian University of Sport Sciences (Budapest, Hungary). The rats were housed in temperature‐controlled animal rooms maintained on a 12:12‐h light–dark cycle. The animals were fed *ad libitum* on a standard chow diet and water. This study was conducted in accordance with the requirements of The Guiding Principles for Care and Use of Animals, EU, and was approved by The National Animal Research Ethical Committee (Hungary) (PE/EA/62‐2/2021).

### 2.2 Maximal Oxygen Uptake Test

All rats were acclimated to a motor‐driven animal treadmill in a closed chamber customized for rats (Columbus Instruments, USA) prior to $\dot{V}$O_2max_ measurements. Briefly, the animals rested on a running belt for 5 min, and the running speed was gently increased. Acclimatization runs were performed for 5–10 min at speeds of 5–25 m/min for two consecutive days. $\dot{V}$O_2max_ was measured in the HCR/LCR rats by beginning the run at a speed of 5 m/min after 5 min of rest and then increasing the speed by 5 m/min every 2 min until exhaustion. The $\dot{V}$O_2max_ was determined for each animal using the following criteria: (i) no change in $\dot{V}$O_2_ with increasing speed, (ii) rats could no longer maintain their posture on the treadmill, and (iii) the respiratory quotient (RQ = $\dot{V}$CO_2_/$\dot{V}$O_2_) > 1 (Marton et al. 2015; Torma et al. 2014). More specifically, $\dot{V}$O_2max_ values were recorded when the animal met one of the three criteria. We also calculated the total running time and total running distance at the time point of $\dot{V}$O_2max_ value onset. Notably, $\dot{V}$O_2max_ measurements were carried out in an infrared light–dark environment, at a temperature of 22°C ± 2°C and a humidity of approximately 55 ± 10%.

### 2.3 Organ Removal and DNA Extractions

After completing the $\dot{V}$O_2max_ measurements, dissection was performed after at least 48 h of rest. More specifically, rats were deeply anesthetized by intraperitoneal injection of a ketamine (Richter, concentration: 100 mg/mL)/xylazine (Produlab Pharma, concentration: 20 mg/mL) cocktail at a dose of 0.1 mL/10 g body weight and intraperitoneally perfused with heparinized ice‐cold saline. The hippocampus, heart, soleus muscle, and large intestine were removed when the rats did not respond to tail compression. Organ samples were stored at −80°C until DNA extraction. One whole side of the hippocampus, left side of the soleus muscle, and middle part of the large intestine, which corresponds to the colon, were used for further analysis. Whole hearts were pre‐homogenized in 2× volume of phosphate‐buffered saline for each organ weight. DNA was extracted using the PureLink Genomic DNA Mini Kit (Thermo Fisher Scientific, USA) according to the manufacturer's instructions. The extracted DNA was dissolved in 50 μL of PureLink Genomic Elution Buffer (10 mM Tris–HCl, pH 9.0, 0.1 mM ethylenediaminetetraacetic acid). Prior to DNA methylation measurement, DNA samples were adjusted to a concentration of ≥ 35 ng/μL with a purity of A260/280 > 1.7.

### 2.4 Global Methylation Measurement by RRBS

RRBS libraries were generated using the Premium RRBS Kit V2 (Diagenode, Seraing, Belgium) as described previously (Veillard et al. 2016). Briefly, 100 ng of isolated DNA was digested with the MspI restriction enzyme, followed by end repair, adaptor ligation, and size selection using Ampure beads. Each library was quantified by quantitative PCR to determine the library concentration, and eight samples were pooled in equimolar amounts using an Excel pooling tool provided by the manufacturer. The pooled samples were subjected to bisulfite treatment, purification, and PCR amplification, according to the manufacturer's instructions. Library concentrations were quantified using the Qubit dsDNA HS Assay (Life Technologies) and library size distribution was measured using a Bioanalyzer High‐Sensitivity DNA chip. The libraries were sequenced by multiplexing eight libraries per lane on an Illumina HiSeq2500 sequencer in 1 × 50 single‐end mode. Sequencing reads were trimmed using CutAdapt to remove adapter sequences. After read trimming, bisulfite alignment to the mRatBN7.2 (GCA_015227675.2) reference sequence and methylation calling were performed using Bismark v0.24.129. We filtered the aligned data to retain only cytosines with > 5× coverage in at least 90% of samples. Methylation values for samples at CpG sites with < 5× coverage were set to Not Available. There were 1,327,821 such highly covered and mostly common CpG sites.

### 2.5 Development of RRBS‐Based Rat Epigenetic Clocks

We developed RRBS‐based rat clocks using an available dataset from the GSE161141 dataset (Levine et al. 2020). First, we downloaded the processed methylation files (CGmaps) for the 134 whole blood samples of male rats ( *Rattus norvegicus* , F344 strain). Then, we lifted the genomic positions from the rn6 to the rn7 genomes using the *liftover* Python package. For further analysis, we used only the CpG sites that were covered by at least five reads in at least 90% of the samples (there were 1,028,452 such highly covered and mostly common CpG sites).

#### 2.5.1 Rat Clock 1

We filled in the missing values using the mean methylation values of the whole data, then trained glmnet (ElasticNet with alpha = 0.5) at 80% and tested it on 20% of the samples (Figure S1A). The performance of the test set was MAE = 4.3 months and *r* = 0.88 (Figure S1A). The clock used 39 CpG sites in total. We applied the clock (Rat clock 1) on our RRBS rat data after selecting the data for highly covered methylation values (≥ 5 reads) and filling missing values by the mean methylation values of the covered clock CpG sites.

#### 2.5.2 Rat Clock 2

First, we found common CpG sites in the two methylation tables (Levine et al.'s (2020) dataset and our rat dataset). In total, 307,337 common CpG sites were identified. We then trained *glmnet* on 80% of the samples from Levine et al.'s (2020) dataset and tested it on 20% using only 307,337 common CpG sites (Figure S1B). The performance of the test set was: MAE = 4.4 months and *r* = 0.88 (Figure S1B). The clock used 29 CpG sites in total. We applied this clock (Rat clock 2) to our rat data, which were restricted to common CpG sites. We filled missing values by the mean methylation values of the covered clock CpG sites. Given the number of samples fulfilling the number of CpG sites used for each Rat clock (39 and 29 sites, respectively), Rat clock 2 had a higher prediction reliability than Rat clock 1.

#### 2.5.3 Rat Intersection Clock

We applied our new epigenetic clock method (Kerepesi and Gladyshev 2023) to 64 rat samples. We trained the intersection clock on the Levine et al. (2020) dataset to obtain a rat clock. Details of the intersection clock are provided in Section 2 of the original study. In this section, we describe it here briefly. The goal of this approach was to maximize the use of CpG sites in the training and test sets. For each test sample, we determined the intersection of CpG sites between the training and test datasets. Subsequently, we restricted the training set and test sample to the intersected CpG sites and used the restricted training set and test sample to train and test clocks by fivefold cross‐validation using ElasticNet (glmnet) (Figure S1C). The mean prediction (MeanPred) of the five final models was used to predict the age of the restricted test sample.

#### 2.5.4 Rat rDNA Clock

we obtained the raw sequence reads (paired‐end) of 134 rat blood DNA methylation samples of the Levine et al. (2020) study from the Sequence Read Archive. Bismark (v0.24.2) with Bowtie2 (v2.4.5) was employed to align bisulfite sequencing reads to the rat rDNA sequence (NR_046239.1). Samtools (v1.17) was used for handling and indexing .bam files. We used Bismark's methylation extraction function with the—cytosine_report option to generate cov files containing methylation percentages at the CpG sites of the rDNA. Then, we merged the cov files into a feature table (Table S7). We applied four imputation techniques—mean, KNN, SoftImpute, and PCA—across 2194 CpG sites of the rDNA. For age prediction, we split the dataset into training (80%) and testing (20%) sets. Using each imputation method, we trained three models: ElasticNet (glmnet v2.2.1), gradient boosting, and random forest (RandomForestRegressor and GradientBoostingRegressor of scikit‐learn v1.3.2). The training and testing were repeated for each imputation technique. The best performing results were achieved by gradient boosting with KNN imputation (MAE = 4.03 months and Pearson *r* = 0.77, see Figure S1D). We processed the 64 samples of the LCR/HCR dataset by Bismark in a similar way as described above. We obtained 2253 CpG sites (see Table S8), and after filtering, we identified 2094 common features between the LCR/HCR and Levine et al.'s dataset. Missing values were imputed by the mean of all methylation percentages.

### 2.6 Global Methylation Analysis (GMM and MME)

We calculated the GMM for each sample as the average methylation level. Furthermore, we calculated MME for each sample as the average methylation entropy. Methylation entropy (Hershey and Lee III 1987) was calculated using the Shannon entropy of each individual CpG site of a sample:

$$\text{Entropy}\left(\mathrm{CpG}\_i\right)=-m\_i\times \log\;\left(m\_i\right)–\left(1–m\_i\right)\times \log\;\left(1–m\_i\right),$$

where m_i is the methylation level of the CpG site and log is the two‐based logarithm.

### 2.7 Promoter Analysis of Individual Genes

We used the methylation table of the 64 rat samples described above (containing only 1,327,821 CpG sites covered by at least five reads in at least 90% of the samples). The promoter region of each gene was determined as [−1500, +500] bp from the transcription start site following the direction of transcription. We used the annotation file GCF_015227675.2_mRatBN7.2_genomic.gtf downloaded from the National Center for Biotechnology Information File Transfer Protocol repository (https://ftp.ncbi.nlm.nih.gov/genomes/all/GCF/015/227/675/GCF_015227675.2_mRatBN7.2/). The mean methylation levels of the CpG sites in the promoter region of each sample were calculated (Table S6).

### 2.8 Gene Enrichment Analysis

Enrichment analysis of gene sets showing significant promoter methylation differences between the LCR and HCR was performed for each organ. The Database for Annotation, Visualization, and Integrated Discovery was used for functional enrichment analysis of significantly different gene sets, focusing on biological processes (Huang et al. 2009; Sherman et al. 2022).

### 2.9 Quantitative Real‐Time PCR

Total RNA was extracted from rat tissues using the RNeasy Mini Kit (Qiagen). Reverse transcription was performed with a Maxima First Strand cDNA Synthesis Kit (Qiagen) for qRT‐PCR, using 39.2–700 μg of RNA per sample, depending on the tissue type. For qRT‐PCR, 1 μL of cDNA was mixed with 5 μL of LightCycler 480 SYBR Green I Master (Roche) and 4 μL of the appropriate primers (Eurofins, see below) using the Bravo pipetting robot (Agilent). All samples were analyzed in technical triplicates, and the average CT values were used to determine relative gene expression via the ΔΔCT method. Fold changes were calculated as the mean fold change relative to the housekeeping genes beta‐actin and HPRT‐1. Two reference genes were used for each qRT‐PCR analysis (ACTB and HPRT). Sequences were:

*ACTB*,

fw: CCCGCGAGTACAACCTTCTTG

rev: TCATCCATGGCGAACTGGTGG

HPRT1,

fw: CTCATGGACTGATTATGGACAGGAC

rev: GCAGGTCAGCAAAGAACTTATAGCC.*Acot5‐ps1*, *Stk24*, *Tuba4a, Sfmbt2*, *Klk5l, hist1h2ail2*, and *Clec2d* expression levels were tested using at least two alternative primer pairs. Sequences were:

*Acot5‐ps1 primer pair 1*,

fw: GGACAATCCCAGCAAAACTGT

rev: ATCCTTCCTCAAACACAGTCCA

*Acot5‐ps1 primer pair 2*,

fw: TACACGGCATTTGCACTACG

rev: CGAGGGATTGGAACCTGGAC

*Stk24 primer pair 1*,

fw: AAGGCATCGACAATCGGACT

rev: CCTCTATCTCGTCCTCCGCT

*Stk24 primer pair 2*,

fw: GGCATCGACAATCGGACTCA

rev: TCCTCTATCTCGTCCTCCGC

*Tuba4a primer pair 1*,

fw: TATGCCCGTGGTCACTACAC

rev: TCCTGTACACTGATCAGACAGC

*Tuba4a primer pair 2*,

fw: AGGGGATGACTCCTTCACCA

rev: GCCATTTCGGATCTCATCAATTACA

*Sfmbt2 primer pair 1*,

fw: ACTGCAAGTTCCTGCAAGGT

rev: CCTTAATTCATCAAACTCCTCTCGG

*Sfmbt2 primer pair 2*,

fw: CAGGAGCCGCCAATGAGAA

rev: TTAAGCACCAGCACGCACTT

*Clec2d primer pair 1*,

fw: GCTTCAGCAAGAAGAAGGTGC

rev: GGCATTTAGTAGGGCCGGTT

*Clec2d primer pair 2*,

fw: ACAGAGAGTCATCAGCGCAC

rev: CTCCTCCGATGGAAACCGAG

*Clec2d primer pair 3*,

fw: AGCAAGAAGAAGGTGCAGAT

rev: GCATTTAGTAGGGCCGGTT

*Clec2d primer pair 4*,

fw: ACAGAGAGTCATCAGCGCAC

rev: CTCCTCCGATGGAAACCGAG

*hist1h2ail2 primer pair 1*,

fw: CGGCGTTCTGCCAAACATC

rev: GAGCCTTTGGTGATCCCTGG

*hist1h2ail2 primer pair 2*,

fw: TTCGTTTCTTTGCTATGTCTGGA

rev: TAGTTGCCCTTACGCAGCAG

*Klk5l primer pair 1*,

fw: CCACAATGAGCACTTCTCCC

rev: GTGTGGGATGGAATGTCGGA

*Klk5l primer pair 2*,

fw: AGAGACACCTGCATGGGTGA

rev: GGGTTACCACCCCATGATGTAA.

### 2.10 Statistical Analysis

Correlations were evaluated by Pearson correlation coefficient (“*r*”) and the corresponding two‐sided *p*‐values using the stats.pearsonr function of the python package scipy (stats module). We used a two‐sided *p*‐value in the study; statistical significance was set at *p* < 0.05. If *p*‐values were indicated by an asterisk, we used the following notations: ns, *p* > 0.05; *, 0.01 < *p* ≤ 0.05; **, 0.001 < *p* ≤ 0.01; ***, *p* ≤ 0.001; and ****, *p* ≤ 1.00e‐04. For the gene enrichment analysis, multiple testing correction was applied using the Benjamini–Hochberg method to calculate the FDR, with significance set at FDR < 0.05.

## 3 Results

### 3.1 Global Changes in the Rat Methylome During Aging Is Delayed in HCR Compared to LCR Rats

**FIGURE 1:**
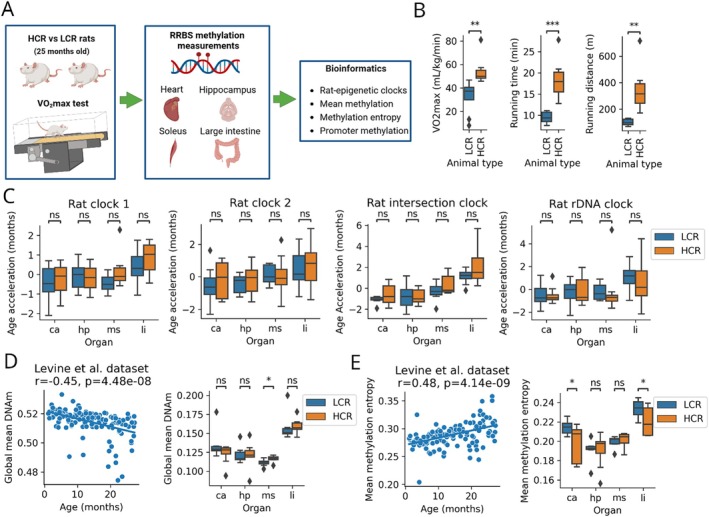
Global changes in the rat methylome during aging is delayed in HCR rats compared to LCR rats. (A) The overview of the study design. (B) Maximal treadmill running capacity of LCR and HCR rats. (C) Epigenetic age accelerations of four rat clocks in multiple organs of LCR and HCR rats. (D) Global DNAm levels of 1,028,452 CpG sites during aging (using the Levine et al. rat blood dataset) and their comparison in multiple organs of LCR and HCR rats. (E) Mean methylation entropy of CpG sites during aging (using the Levine et al. rat blood dataset) and their comparison in multiple organs of LCR and HCR rats. ca, heart; HCR, high‐capacity runner; hp, hippocampus; LCR, low‐capacity runner; li, large intestine; ms, soleus muscle; ns: not significant; *r*, Pearson's correlation coefficient. *p*, *P* value of each test, *: 0.01 < *p* ≤ 0.05, **: 0.001 < *p* ≤ 0.01, ***: *p* ≤ 0.001.

To determine the relationship between differences in CRF, epigenetic age, and global changes in the methylome with aging across multiple organs, we obtained DNA methylome data from the hippocampus, heart, soleus muscle, and large intestine of 16 (LCR: *n* = 8 and HCR: *n* = 8) 23–24 months old rats, which were selectively bred for 44 generations based on endurance running capacity (Koch and Britton 2001) (Figure 1A and Table 1). At least 48 h before organ sample collection, the maximum treadmill running capacity of LCR and HCR rats was measured, which revealed that CRF total running time and total running distance were higher in the HCR group than in the LCR group. These results indicated that CRF, which gradually declines with age, retains differences in these rat models even at this age (Figure 1B). Genomic DNA extracted from each organ sample was subjected to RRBS, and we obtained data for > 6 million CpG sites in the 64 samples.

**TABLE 1:** Chronological age, body, and organ weight of LCR and HCR rats.

	LCR (*n* = 8)	HCR (*n* = 8)	*p*
Age (months)	25.3 ± 0.3	25.4 ± 0.4	0.888
Body weight (g)	312 ± 40	277 ± 50	0.154
Hippocampus (mg)	59 ± 5	58 ± 4	0.430
Heart (mg)	933 ± 79	926 ± 53	0.830
Soleus (mg)	99 ± 16	108 ± 16	0.297
Large intestine	N.A.	N.A.	—

*Note:* Age indicates the chronological age at the time of the dissection. Organ weights are not shown for the large intestine, as part of it was cut off and stored. LCR: low‐capacity runner, HCR; high‐capacity runner, N.A: not applicable.

Abbreviations: HCR, high‐capacity runner; LCR, low‐capacity runner; N.A, not applicable.

To develop an RRBS‐based rat epigenetic clock of our rat data, we downloaded the processed methylation files (CGMap) of the GSE161141 dataset (Levine et al. 2020) and developed “Rat clock 1” and “Rat clock 2” (see details in Section 2). Both clocks were trained on 80% (*n* = 107) of the random samples using the machine‐learning tool glmnet and tested on 20% of the samples (*n* = 27). The performance on the test set was mean absolute error (MAE) = 4.3 months and *r* = 0.88 by Rat clock 1 and MAE = 4.4 months and *r* = 0.88 by Rat clock 2 (Figure S1A,B). A total of 39 CpG sites were used by Rat clock 1 (Table S4), and 29 CpG sites were used by Rat clock 2 (Table S5). Age acceleration was calculated from the residuals of the regression line on the test set for the two rat clocks. Owing to the random nature of RRBS, the number of clock CpG sites in the application dataset may be limited. Therefore, we found common CpG sites in the training dataset (i.e., the Levine et al. rat data) and the samples of the application dataset (i.e., our rat data) and developed a ‘Rat intersection clock,’ on the common CpG sites according to the intersection clock method (Kerepesi and Gladyshev 2023, also see Figure S1C). Furthermore, we developed a rat ribosomal DNA (rDNA) clock again using the 134 blood samples of the Levine et al. rat dataset (see Section 2). Its performance on the test set was MAE = 4 months and *r* = 0.77 (Figure S1D). The benefit of the rDNA clock is that the multiplicity of the rDNA in the genome leads to higher read coverage of the CpG sites resulting in an improved applicability on external datasets. The top features of the rat rDNA clock were the CpG sites that showed strong positive correlation with age. Interestingly, there was no strong negative correlation with age among the CpGs sites of the rat rDNA (Figure S1E,F). As we applied these four rat clocks to our dataset, we unexpectedly observed no difference between the LCR and HCR groups in the acceleration of the age of the rat clocks in any organ (Figure 1C).

We also used the Levine et al. rat blood dataset to test the association between the global mean DNA methylation (GMM), mean methylation entropy (MME), and aging. We found that GMM was negatively correlated (*r* = −0.45; *p* = 4.48e‐08) (Figure 1D) and MME was positively correlated (*r* = 0.48; *p* = 4.14e‐09) (Figure 1E) with age. We then compared the GMM and MME in each organ between the LCR and HCR groups. GMM in the LCR soleus muscle samples was lower than that in the HCR samples (Figure 1D). The MME was higher in the LCR heart and large intestine samples than in the HCR samples (Figure 1E).

Altogether, while the applied blood‐based epigenetic clocks did not show age acceleration differences between LCR and HCR rats for any of the examined organs, we observed that the HCR rat methylome as a whole expressed a younger state compared to the LCR rat methylome.

### 3.2 LCR and HCR Rat Had the Top Hit Genes in the Soleus Muscle and Seven Common Genes Across All Four Organs

**FIGURE 2:**
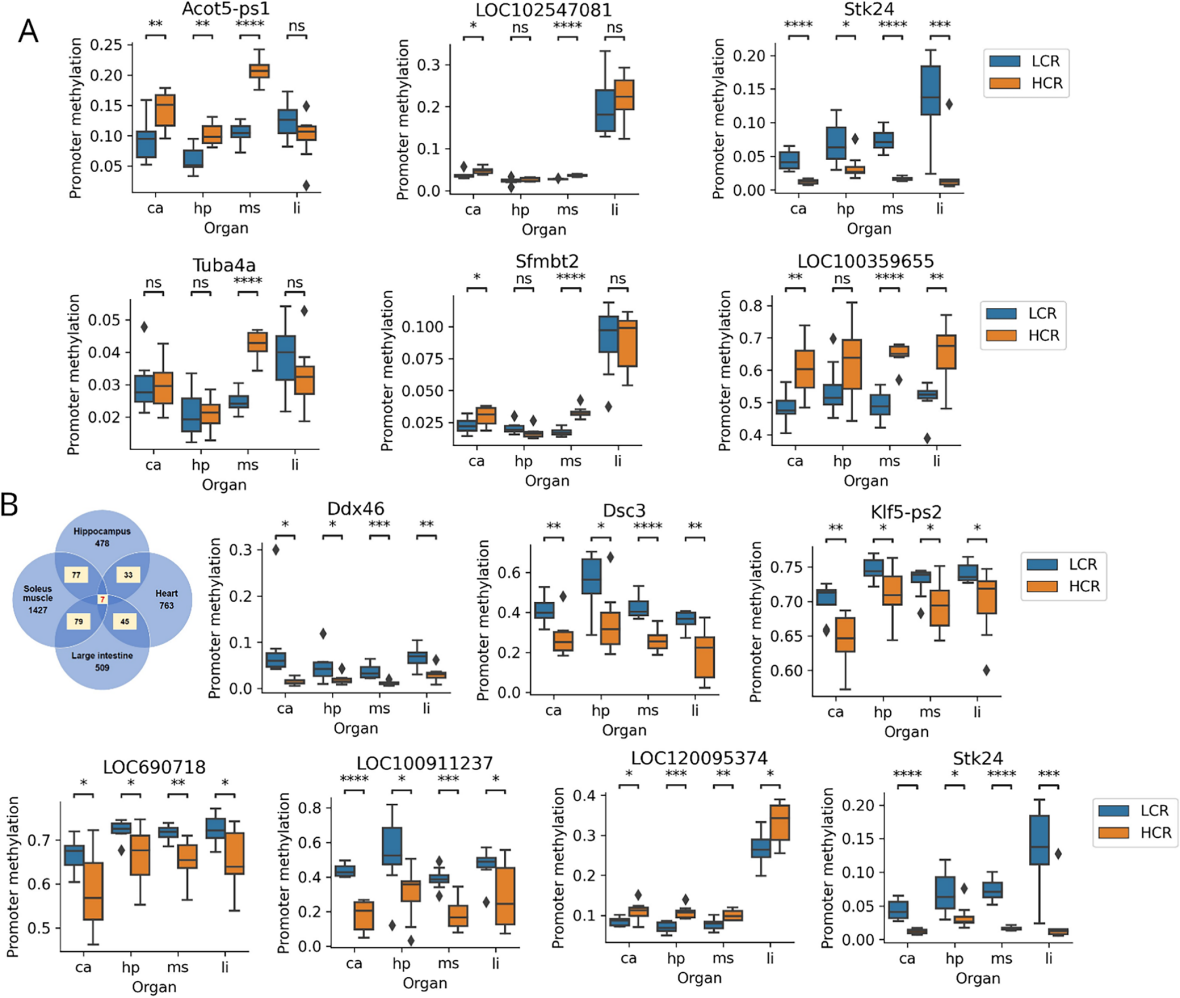
Organ commonality of promoter methylation in individual genes in LCR and HCR rats. (A) Six best hit genes across the four targeted organs of LCR and HCR rats. (B) The seven common genes influenced in promoter methylation by CRF across four organs. ca, heart; HCR, high‐capacity runner; hp, hippocampus; LCR, low‐capacity runner; li, large intestine; ms, soleus muscle; ns: not significant. *Acot5‐ps1*: Acyl‐CoA Thioesterase 5 Pseudogene 1, *Stk24*: Serine/Threonine Kinase 24, *Tuba4a*: Tubulin Alpha 4a, *Sfmbt2*: Scm‐Like with Four Mbt Domains 2, *LOC102547081/LOC100359655*: uncharacterized genes. *Ddx46*: DEAD‐Box Helicase 46, *Dsc3*: Desmocollin‐3, *Klf5‐ps2*: Kruppel‐Like Factor 5 Pseudogene 2, *LOC690718/LOC100911237/LOC120095374*: Uncharacterized genes. *: 0.01 < *p* ≤ 0.05, **: 0.001 < *p* ≤ 0.01, ***: *p* ≤ 0.001, ****: *p* ≤ 0.0001, ns: not significant.

To identify the best‐hit genes and common genes affected by CRF‐induced promoter methylation across all four organs, we calculated the mean methylation levels of CpG sites in the promoter region of each sample. Among the 14,366 examined genes, we identified the six best‐hit genes with the lowest *p*‐values across all four organs in the LCR and HCR groups. These best‐hit genes were *Acot5‐ps1* (Acyl‐CoA Thioesterase 5 Pseudogene 1; *p* = 5.813623e‐08, *p_bonf* = 0.000835), *LOC102547081* (uncharacterized gene; *p* = 1.517911e‐07, *p_bonf* = 0.002181), *Stk24* (Serine/Threonine Kinase 24, *p* = 3.676357e‐07, *p_bonf* = 0.005281), *Tuba4a* (Tubulin Alpha 4a; *p* = 7.127704e‐07, *p_bonf* = 0.01024), *Sfmbt2* (Scm‐Like with Four Mbt Domains 2; *p* = 1.774531e‐06, *p_bonf* = 0.025493), and *LOC100359655* (uncharacterized gene; *p* = 3.207110e‐06, *p_bonf* = 0.046073), all of which were observed in the soleus muscle with an uncorrected *p*‐value lower than 3.208E‐06 and Bonferroni corrected *p* < 0.0461 (Figure 2A). Pseudogenes, such as *Acot5‐ps1*, do not typically have a functional role and are non‐coding DNA sequences. *Stk24* is a serine/threonine kinase involved in signal transduction pathways that influence cellular processes associated with growth, differentiation, and various physiological functions. *Tuba4a* encodes the tubulin protein, which is a structural component of microtubules. Microtubules are essential for various cellular processes, including cell division, intracellular transport, and cell shape maintenance. *Sfmbt2* is associated with epigenetic regulation, involved in the maintenance of chromatin structure, and plays a role in controlling gene expression and cellular development. *LOC102547081* and *LOC100359655* are uncharacterized genes and their precise physiological roles remain unknown.

We also identified a distinct set of seven genes that exhibited consistently significant (*p* < 0.05) differential methylation levels across all four organs examined in the LCR and HCR groups (Figure 2B). The seven identified common genes comprised *Ddx46* (DEAD‐Box Helicase 46), *Dsc3* (Desmocollin‐3), *Klf5‐ps2* (Kruppel‐Like Factor 5 Pseudogene 2), *LOC690718* (uncharacterized gene), *LOC100911237* (uncharacterized gene), *LOC120095374* (uncharacterized gene), and *Stk24* (Serine/Threonine Kinase 24), and except for *LOC120095374*, hypomethylation was observed in the HCR group when compared to the LCR group across all four organs (Figure 2B). *Ddx46* is an RNA helicase involved in RNA processing and translational regulation. *Dsc3* is a component of desmosomes that plays a critical role in cell–cell adhesion, contributing to tissue integrity and cohesion. *Klf5‐ps2* is a transcription factor implicated in the regulation of cell proliferation, differentiation, and growth. *Stk24* is a serine/threonine kinase associated with various cellular functions including signal transduction and cell growth regulation. The precise physiological roles of *LOC690718*, *LOC100911237*, and *LOC120095374* are yet to be elucidated. Therefore, further investigation is required to determine the specific functions of these genes.

### 3.3 LCR and HCR Rats Had Different Methylation Levels in Gene‐Specific Promoter Regions Depending on Organs

**FIGURE 3:**
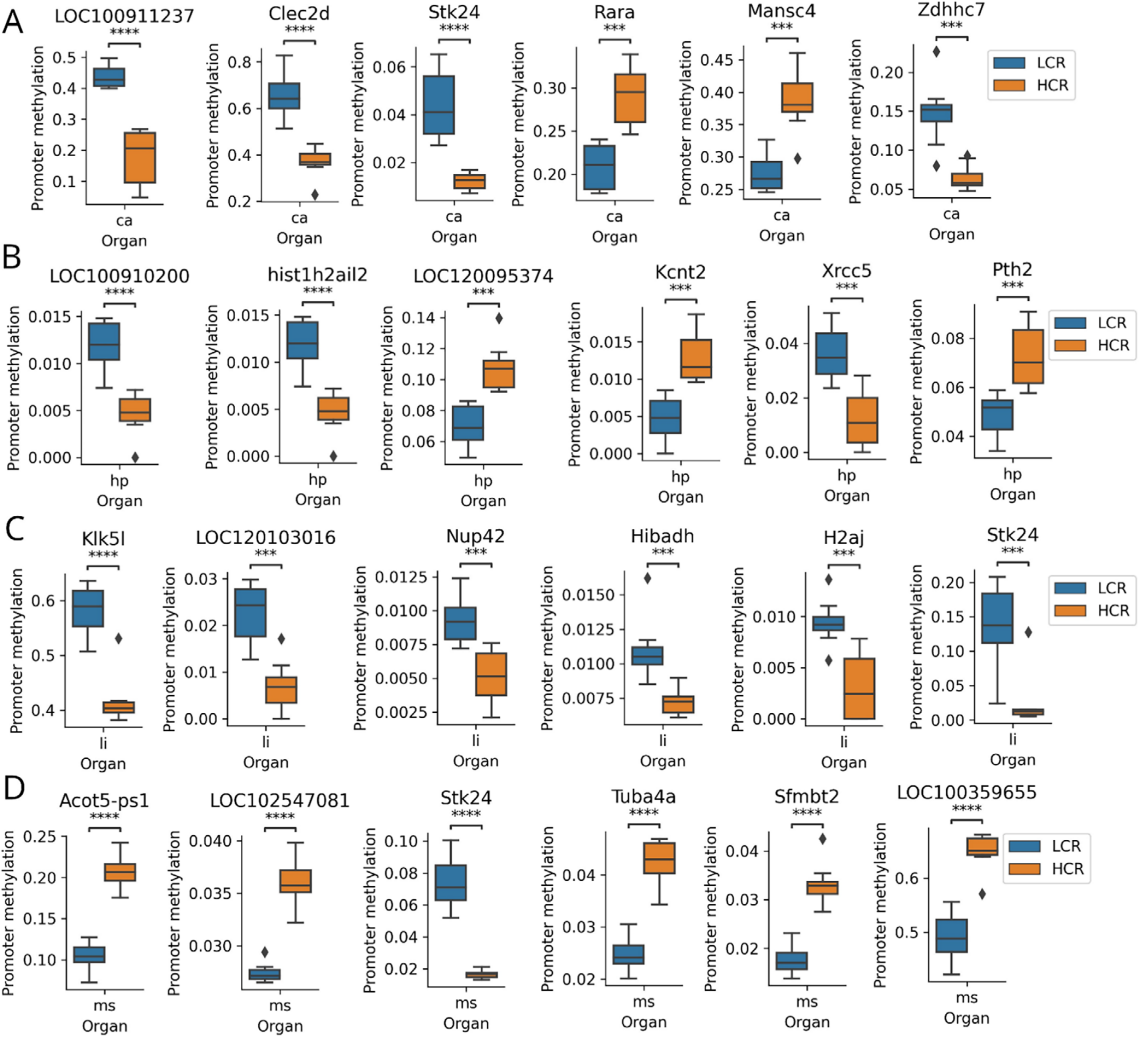
Organ specificity of promoter methylation in individual genes in LCR and HCR rats. Six best hit genes influenced in promoter methylation in the (A) Heart, (B) Hippocampus, (C) Large intestine, and (D) Soleus muscle. ca, heart; HCR, high‐capacity runner; hp, hippocampus; LCR, low‐capacity runner; li, large intestine; ms, soleus muscle; ns: not significant. *Clec2d*: C‐type lectin domain family 2, member D, *Stk24*: Serine/Threonine Kinase 24, *Rara*: Retinoic Acid Receptor Alpha, *Mansc4: MANSC Domain Containing 4*, *Zdhhc7*: Zinc Finger DHHC‐Type Containing 7, *hist1h2ail2*: Histone Cluster 1 H2A Family Member L2, *Kcnt2*: Potassium Channel, Sodium‐Activated, Subfamily T, Member 2, *Xrcc5*: X‐Ray Repair Cross Complementing 5, *Pth2*: Parathyroid Hormone 2, *Klk5l*: Kallikrein‐Related Peptidase 5‐Like, *Nup42*: Nucleoporin 42, *Hibadh*: 3‐Hydroxyisobutyrate Dehydrogenase, *H2aj*: Histone H2AJ, *Acot5‐ps1*: Acyl‐CoA Thioesterase 5 Pseudogene 1, *Tuba4a*: Tubulin Alpha 4a, *Sfmbt2*: Scm‐Like with Four Mbt Domains 2. *LOC100911237*, *LOC100910200*, LOC120095374, *LOC120103016, LOC102547081*, and *LOC100359655* are currently uncharacterized genes. ***: *p* ≤ 0.001, ****: *p* ≤ 0.0001.

Next, we identified gene characteristics of each organ with different methylation levels in their promoter regions. We used the data on the mean methylation level at the CpG site of the promoter region of each sample, calculated by the method described above, and illustrated the six best‐hit genes for each organ with the lowest *p*‐value in the comparison between the LCR and HCR groups (Figure 3A–D).

In the heart, the best‐hit genes were *LOC100911237* (uncharacterized gene; *p* = 0.000005), *Clec2d* (C‐type lectin domain family 2, member D; *p* = 0.000012), *Stk24* (Serine/Threonine Kinase 24; *p* = 0.000031), *Rara* (Retinoic Acid Receptor Alpha; *p* = 0.00012), *Mansc4 (Mansc4; p* = 0.000143), and *Zdhhc7* (Zinc Finger DHHC‐Type containing 7; *p* = 0.000159) (Figure 3A). *Clec2d* is associated with the immune system and possibly plays a role in immune responses and cell–cell interactions, particularly in the context of immunity and defense against pathogens. *Stk24* is a serine/threonine kinase that is involved in signal transduction pathways, influences cellular processes related to growth and differentiation, and potentially plays a role in various physiological functions. *Rara* is a nuclear receptor that responds to retinoic acid, a form of vitamin A, which plays a crucial role in regulating gene expression and is involved in processes such as cell differentiation and development. Information regarding the specific physiological role of *Mansc4* is limited, and further research is required to elucidate its function. *Zdhhc7* encodes a protein with a DHHC domain potentially involved in palmitoylation, which can affect the function and localization of various proteins, including those involved in cell signaling.

The best‐hit genes in the hippocampus were *LOC100910200* (uncharacterized gene; *p* = 0.000048), *hist1h2ail2* (Histone Cluster 1 H2A Family Member L2; *p* = 0.000048), *LOC120095374* (uncharacterized gene; *p* = 0.000137), *Kcnt2* (Potassium Channel, Sodium‐Activated, Subfamily T, Member 2; *p* = 0.000199), *Xrcc5* (X‐Ray Repair Cross Complementing 5; *p* = 0.000261), and *Pth2* (Parathyroid Hormone 2; *p* = 0.0009) (Figure 3B). The *hist1h2ail2* protein is involved in DNA packaging and gene regulation and is possibly associated with chromatin structure and epigenetic regulation. *Kcnt2* encodes a sodium‐activated potassium channel that regulates the electrical activity of cells and is involved in ion transport and cellular excitability. *Xrcc5* is involved in DNA repair and associated with the maintenance of genomic stability and repair of DNA damage caused by various factors. *Pth2* encodes a parathyroid hormone that plays a role in calcium and phosphate homeostasis, thereby influencing bone health and mineral metabolism.

In the large intestine, the best‐hit genes were *Klk5l* (Kallikrein‐related peptidase 5‐like; *p* = 0.000005), *LOC120103016* (uncharacterized gene; *p* = 0.000147), *Nup42* (nucleoporin 42; *p* = 0.000531), *Hibadh* (3‐hydroxyisobutyrate dehydrogenase; *p* = 0.000668), *H2aj* (histone H2AJ; *p* = 0.000826), and *Stk24* (serine/threonine kinase 24; *p* = 0.00094) (Figure 3C). *Klk5l* is possibly a member of the kallikrein‐related peptidase family, which includes enzymes involved in various physiological processes such as tissue remodeling, inflammation, and blood pressure regulation. *Nup42* is a nucleoporin component of the nuclear pore complex that regulates the transport of molecules between the nucleus and cytoplasm. *Hibadh* is involved in fatty acid metabolism and the breakdown of 3‐hydroxyisobutyrate, a metabolite associated with valine catabolism and energy production. The *H2aj* protein is involved in DNA packaging and gene regulation and may play a role in chromatin structure and epigenetic regulation. *Stk24* is a serine/threonine kinase involved in signal transduction pathways that influence cellular processes associated with growth, differentiation, and various physiological functions.

The best hit genes in soleus muscle were *Acot5‐ps1*, *LOC102547081*, *Stk24*, *Tuba4a*, *Sfmbt2*, and *LOC100359655* (Figure 3D), which have been described above.

We also performed enrichment analysis focusing on gene sets that showed significant promoter methylation differences between LCR and HCR in each organ (Figure S2). The analysis revealed that only three biological processes in the soleus muscle—transcription, transcription regulation, and neurogenesis—reached the significance threshold (*p* < 0.05) after Benjamini–Hochberg correction. In contrast, no biological processes in the heart, hippocampus, and large intestine exhibited a corrected *p*‐value below 0.05. These results indicate that the soleus muscle is particularly susceptible to CRF, demonstrating significant involvement of gene regulation‐related biological processes.

### 3.4 Gene Expression Analysis of the Top Differently Methylated Genes Between LCR and HCR Rats

We performed quantitative real‐time PCR (qRT‐PCR) experiments to evaluate the gene expression of the top differentially methylated genes between LCR and HCR rats for each organ. No detectable expression was observed for *Acot5‐ps1*, *Sfmbt2*, *Klk5l*, and *Clec2d* across all tissues. Clear expression of *Stk24* was detected using two primer pairs; no significant differences were observed in the soleus muscle (Figure S4A), whereas *Stk24* expression was significantly higher in the heart of HCR rats compared to LCR rats (Figure S4B). Expression of *Tuba4a* (Figure S4C) and *Hist1h2ail2* (Figure S4D) showed no significant differences in the soleus muscle and the hippocampus.

### 3.5 Age Acceleration of the Soleus Muscle Was Significantly Higher Compared to the Heart and the Hippocampus, and Significantly Lower Compared to the Large Intestine

**FIGURE 4:**
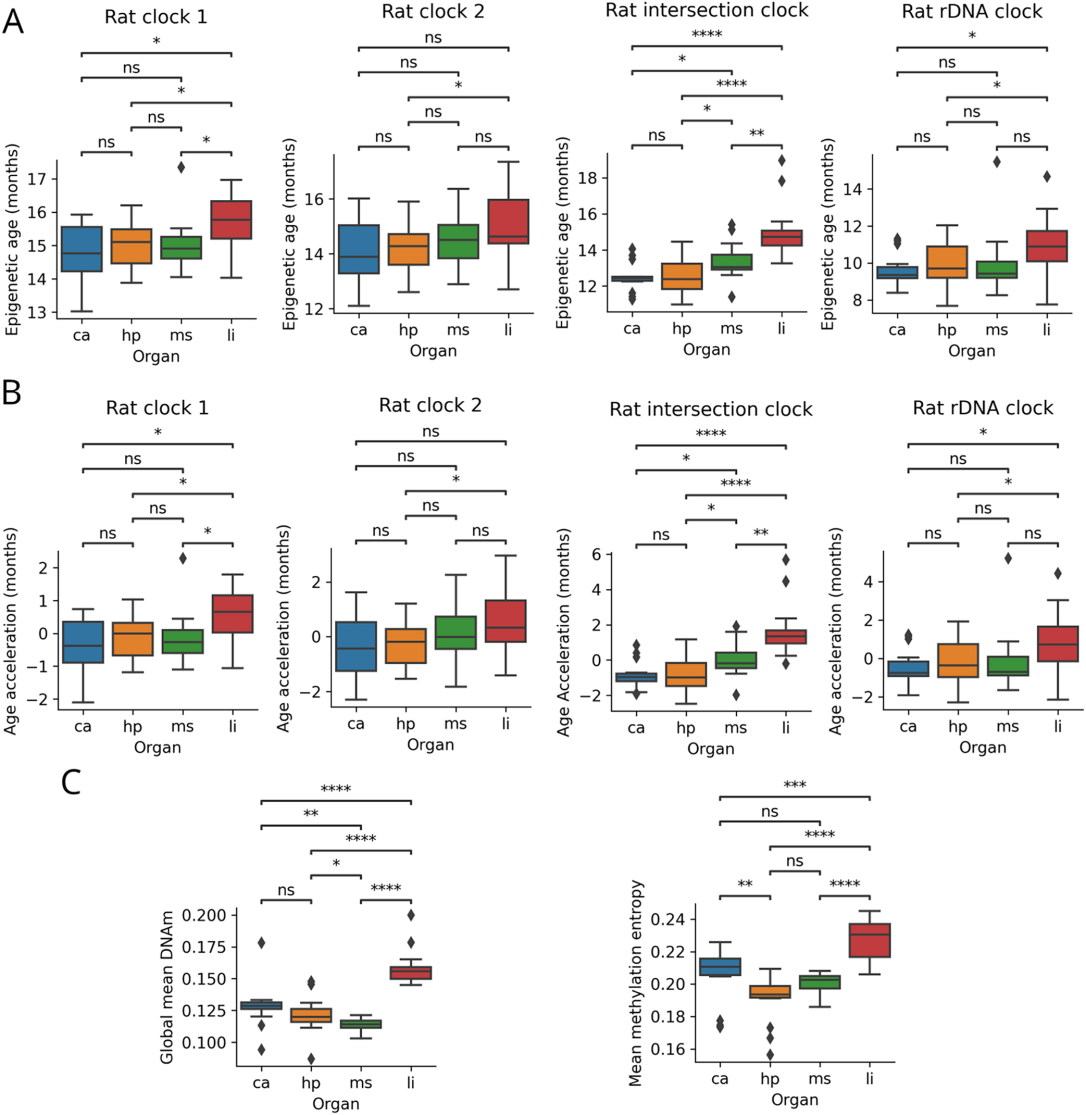
Multi‐organ comparisons of epigenetic aging considering all of the 64 samples including LCR and HCR rats. (A) Multi‐organ comparisons of epigenetic age of four rat clocks. (B) Multi‐organ comparisons of epigenetic age acceleration of four rat clocks. (C) Multi‐organ comparisons of global mean DNAm level and mean methylation entropy. ca, heart; hp, hippocampus; li, large intestine; ms, soleus muscle. *: 0.01 < *p* ≤ 0.05, **: 0.001 < *p* ≤ 0.01, ***: *p* ≤ 0.001, ****: *p* ≤ 0.0001, ns: not significant. Paired *t* tests were applied.

In addition to the comparisons between the LCR and HCR groups, we compared the rates of epigenetic aging between organs using data from 16 samples from both the LCR and HCR groups. The epigenetic age and age acceleration according to the Rat clock 1 were significantly higher in the large intestine than in the heart, hippocampus, and soleus muscle (Figure 4A,B). The Rat clock 2 showed significantly higher epigenetic age and age acceleration of the large intestine compared to the hippocampus (Figure 4A,B). Using the Rat intersection clock, that is expected to be the most robust and reliable method, the epigenetic age and age acceleration were significantly higher in the large intestine than in the heart, hippocampus, and soleus muscle. Additionally, the epigenetic age and age acceleration of the soleus muscle was significantly higher compared to the heart and the hippocampus (Figure 4A,B). The rat rDNA clock showed significantly higher epigenetic age and age acceleration of the large intestine compared to the heart and hippocampus (Figure 4A,B). GMM was significantly higher in the large intestine than in the heart, hippocampus, and soleus muscle, and it was significantly lower in the soleus muscle compared to the heart and hippocampus (Figure 4C). MME was significantly higher in the large intestine than in the heart, hippocampus, and soleus muscle, and was significantly higher in the heart compared to the hippocampus (Figure 4C). Overall, the set of epigenetic aging measurements in the large intestine and soleus muscle showed different signatures compared to the other two organs. The most remarkable and consistent finding is that the large intestine is epigenetically older compared to the remaining organs.

### 3.6 Inconsistency of Epigenetic Age Acceleration Across Organs Within the Same Individual

As we measured the epigenome of four organs per individual, we had the unique opportunity to investigate whether accelerated (or decelerated) aging is a global process affecting the whole body or rather appears only locally for a specific organ. We found no significant correlations between any organ pair, meaning that epigenetic age acceleration is not consistent across organs (Figure S3).

## 4 Discussion

Epigenetic clocks are a promising aging biomarker that can estimate biological age across organs based on age‐related changes in DNA methylation patterns, and have demonstrated high predictive power for age‐related disease onset and mortality in humans (Horvath and Raj 2018; Oblak et al. 2021). Epigenetic clocks have also been developed in rodents such as mice (Meer et al. 2018; Petkovich et al. 2017; Stubbs et al. 2017; Thompson et al. 2018; Wang and Lemos 2019) and rats (Kerepesi et al. 2022; Levine et al. 2020) and pan‐species clocks have been proposed, including the human‐rat pan‐tissue clock (Horvath et al. 2024; Lu et al. 2023). These findings and the latest studies (Crofts et al. 2024) suggest that age‐related changes in DNA methylation patterns are a common mechanism of aging across mammals and, simultaneously, provide a useful tool for evaluating the effectiveness of intervention strategies to delay aging in a relatively short timescale. Epigenetic age can be delayed by caloric restriction, diet quality, growth hormone receptor knockout, plasmapheresis, and lifestyle interventions in rodents and humans (Fahy et al. 2019; Fiorito et al. 2021; Fitzgerald et al. 2021; Meer et al. 2018; Petkovich et al. 2017; Sae‐Lee et al. 2018; Stubbs et al. 2017; Thompson et al. 2018; Wang and Lemos 2019; Waziry et al. 2023; Zhang et al. 2023). Cross‐sectional studies in human populations have shown that physical activity and CRF are associated with delayed epigenetic age progression (Fox et al. 2023; Jokai et al. 2023; Kawamura et al. 2024; Quach et al. 2017). In mice, late‐life exercise training may delay skeletal muscle epigenetic aging via skeletal muscle‐specific epigenetic clock(s) (Jones et al. 2023; Murach et al. 2022). Based on these findings, we applied four rat epigenetic clocks to our LCR/HCR rat dataset. Among them, we determined the genes and gene features associated with the CpG sites of Rat clocks 1 and 2 (Tables S9, S10), aside from the complex intersection clock containing 5 × 64 clocks and the rDNA clock developed from the CpG sites of rDNA. Our data provide valuable insight into the relationship between DNA methylation and aging, as shared genes such as *GDF11* were identified in both clocks; *GDF11* has been actively discussed in relation to aging and deserves attention (Sinha et al. 2014). In contrast to the previous studies, we demonstrated that blood‐based rat epigenetic clocks do not consistently reflect differences in CRF across all organs. These findings may have resulted from the difference in experimental design between the two studies, which compared two groups of mice with 8 weeks of voluntary endurance/resistance exercise training and the present study, which compared groups of rats with different CRF that did not undergo regular exercise training. In addition, this result may be partly explained by the fact that the rat clocks were based on whole blood samples and that our dataset consisted of organ samples. However, in previous studies, blood‐based RRBS clocks successfully captured rejuvenation effects in other tissues and cells (Kerepesi et al. 2021; Petkovich et al. 2017; Zhang et al. 2023). Another possible limitation of our analysis is that the training set of the rat clocks contained only males while our dataset contained only females. This may influence the accuracy of the clocks and explain the lower predicted age compared to the chronological age (Figure 4A). The tissue‐specific analyses of another study using the same rat model but the mammalian methylation array platform revealed a lower epigenetic age (adjusted for chronologic age, generation, sex and treadmill running distance at 3 months of age) in HCR rats for adipose, skeletal muscle, cardiac muscle (pan‐tissue rat clock; *p <* 0.04), and liver (human‐rat clock for relative and chronologic age; *p* < 0.01) compared to LCR rats (Rossiter et al. 2023). However, they did not examine the hippocampus, soleus muscle, and large intestine. The study also demonstrated that the effect of CRF on epigenetic age was strongest in young rats compared to old rats; therefore, we cannot exclude the possibility that the effect of CRF may be reduced in old rats (Rossiter et al. 2023). Finally, the study measured male and female rats, while we measured only females. It is possible that the effect of high CRF on the rate of aging is milder in females compared to males. This would be consistent with our recent study where we found that standard blood test‐based age acceleration largely decreased in male athletes compared to the healthy male controls but did not significantly decrease in female athletes compared to healthy female controls (Juhász et al. 2024). Another previous study has shown that the age acceleration in the high‐fit group was significantly lower than in the medium‐ to low‐fit group for both men and women, but the difference was slightly lower for women (Jokai et al. 2023). Our previous study also found a significant negative relationship between CRF and age acceleration, but this study included only men (Kawamura et al. 2024). Most other previous studies have examined the relationship between physical activity and age acceleration in the same cohort of men and women (adjusted for sex) through cross‐sectional designs (Quach et al. 2017). Given the findings of previous studies that showed sex differences in the relationship between physical activity and healthspan (Arem et al. 2015; Ji et al. 2024), it is also possible that there are sex differences in the relationship between physical fitness and epigenetic aging. Taken together, the relationship between exercise and multi‐organ epigenetic age is worth investigating, along with the development of a rat clock that can capture biological aging with high precision across multiple organs, as well as exercise training interventions that take into account the mode, intensity, duration, and frequency should also be investigated. In addition, future comparisons of epigenetic aging in LCR/HCR rats at several life stages and between sexes will be needed to elucidate the relationship between CRF and trajectories of epigenetic aging in both males and females.

Measurements that may capture changes in DNA methylation during aging include GMM and MME. Although evidence suggests that GMM generally declines with age, the relationship between these factors appears to vary among organs and measurement methods (Seale et al. 2022). Results from blood‐based methylation assays indicated a negative correlation between GMM and age in mice (Sziráki et al. 2018); however, this relationship was unknown in rats. In this study, we showed for the first time in rats that a negative correlation exists between blood GMM and age and that high CRF mitigates the age‐related decline in GMM only in the rat soleus muscle. Entropy is a measure of disorder in an aging system, in which death is the maximum disorder (Hershey and Lee III 1987). MME has been shown to increase during aging in mice, naked mole‐rats, and humans (Hannum et al. 2013; Kerepesi et al. 2022; Sziráki et al. 2018); however, the relationship between the MME and aging in rats remains unknown. We found a positive correlation between MME and age, and high CRF mitigated age‐related increases in MME in the rat heart and large intestine. These findings on GMM and MME suggest that maintaining high CRF levels may delay age‐related changes in DNA methylation patterns in an organ‐specific manner. A previous study showed that acute exercise decreases global promoter methylation in human skeletal muscles (Barrès et al. 2012). Chronic exercise also affects global promoter methylation in human skeletal muscles (Voisin et al. 2024). However, no study has investigated the relationship between the GMM, MME, and CRF in multiple organs. Considering these facts, the results of this study may provide an insight into the mechanism of the systemic “geroprotective” effect of exercise. However, GMM and MME are not direct measures of epigenetic age, and the differences between the LCR and HCR groups were organ‐specific and inconsistent between GMM and MME, that is, there were group differences in the soleus muscle for GMM, while in the heart and large intestine for MME. Therefore, further studies on the relationship between epigenetic aging and CRF are needed to clarify their organ specificity and organ commonality.

The results of the promoter analysis of individual genes suggest that the soleus muscle methylome is more strongly and extensively affected by CRF than other organs known to be affected by exercise, such as the hippocampus, heart, and large intestine. In contrast, our results identified common genes that showed differences in promoter methylation levels across all four organs between the LCR and HCR groups, as well as top‐hit genes that differed between organs. Enrichment analysis revealed that transcription and transcription regulation were consistently enriched biological processes across all organs; however, adjusted FDR showed significant processes only in the soleus muscle, including transcription, transcription regulation, and neurogenesis. This suggests that while there are shared regulatory mechanisms influenced by CRF, the functional impact on DNA methylation exhibits organ‐specific variation. In addition to this result, the number of organ‐specific genes whose promoter methylation is affected by CRF exceeds the number of organ‐common genes, indicating that the relationship between CRF and DNA methylation levels is partially common across organs but tends to be more organ‐specific. Although most studies examining the relationship between exercise and DNA methylation have focused on skeletal muscle (Bittel and Chen 2024), it is well known that the health‐promoting effects of exercise extend throughout the whole body (Ashcroft et al. 2024; Hawley et al. 2014; Chow et al. 2022). A recent study using a multi‐omics analysis investigated the multi‐tissue molecular response to endurance exercise training and identified 22 genes common to skeletal muscle, white adipose tissue, liver, heart, lung, and kidney, while many of the training‐responsive genes are tissue‐specific (MoTrPAC Study Group 2024). The present study differs from this previous study in its design because we did not subject the animals to endurance exercise training; our results are in line with this previous study in that the effects of high CRF on DNA methylation profiles tend to be organ specific. Additionally, our study evaluated the top differentially methylated gene promoters in LCR/HCR rats. Consistent with the promoter methylation changes, *Stk24* expression was significantly higher in the heart of HCR rats compared to LCR rats. Surprisingly, we have not found significant gene expression changes in the remaining three examined cases. Our data suggest that changes in DNA methylation may influence gene expression processes; however, to fully elucidate the functional significance of these methylation alterations, future studies incorporating more comprehensive transcriptomic and proteomic analyses will be essential. Furthermore, the relationship between CpG methylation and changes in gene expression has been suggested to be stronger for endurance exercise than for resistance exercise (Bittel and Chen 2024); it would be worthwhile to elucidate organ‐specific and/or organ‐common regulatory mechanisms of DNA methylation patterns and their organ interactions in exercise adaptation through endurance exercise interventions. In addition, further validation in both sexes is warranted, as some studies suggest that skeletal muscle methylation responses to exercise training may differ between sexes, although a consensus has yet to be reached (Lindholm et al. 2014; Landen et al. 2023).

Notably, statistical analysis revealed that epigenetic age, epigenetic age acceleration, GMM, and MME exhibited different signatures among the four organs targeted in this study. More specifically, both epigenetic age and epigenetic age acceleration values were highest in the large intestine, and the values of the intersection clock were highest in the large intestine, followed by the soleus muscle. GMM and MME exhibited higher values in the large intestine than in the heart, hippocampus, and soleus muscle. Moreover, GMM and MME values were second highest in the heart. Furthermore, epigenetic age acceleration was not associated across organs within the same individual. Recent results from animal studies using proteomics and transcriptomics suggest that the rate of aging varies not only between individuals but also between organs within individuals (Schaum et al. 2020; Tabula Muris Consortium 2020), consistent with our results on organ distinctions in epigenetic aging. Similar studies have been reported in humans (Oh et al. 2023), and part of the multi‐organ aging network has also been elucidated, in which biological aging in specific organs influences the progression of aging in other organs (Tian et al. 2023). On the other hand, epigenomics‐based studies suggest that age‐related DNA methylation changes are organ‐specific in rodents (Thompson et al. 2010) and humans (Slieker et al. 2018), but the differences in epigenetic age acceleration among organs are largely unknown. Therefore, our findings provide important insights into the biology of aging and emphasize the need to validate rejuvenation strategies in the context of the organ‐specific nature of epigenetic aging. Further evidence is required to elucidate the organ‐specific effects of exercise on organ‐specific epigenetic aging. In addition, each of the four clocks we used may capture a different aspect of aging due to differences in their developmental processes. As noted above, our data consistently showed the highest values in the large intestine. However, given the slight differences in values between clocks in the same organ, the assessment of biological aging using multiple clocks would require the integration of all available data and careful biological interpretation of the clocks.

## 5 Conclusions

In conclusion, while the applied blood‐based rat epigenetic clocks do not consistently reflect the differences in CRF in any organ, higher CRF is associated with a younger state according to GMM and MME. Our results also indicate that CRF regulates promoter methylation of various genes in an organ‐specific and organ‐common manner. We also demonstrated that epigenetic aging exhibits different signatures in different organs and that they are not consistent across organs. These findings emphasize the potential involvement of CRF in organ‐specific epigenetic aging and gene‐specific regulation of promoter methylation, providing novel insights into the complicated interplay between CRF, epigenetic regulation, and aging processes.

## Author Contributions

Conceptualization: T.K. and Z.R. Methodology, T.K., C.K., and Z.R. Investigation: T.K., C.K., F.T., Z.B., L.Z., P.B., A.K., V.P., K.P., E.K., and Z.R. Bioinformatics and statistical analyses: C.K. J.P.S. developed the rat rDNA clock with the supervision of C.K. Writing – original draft: T.K., C.K., and Z.R. Writing – review and editing: T.K., C.K., L.B., M.H., L.G.K., S.L.B., J.P.S., and Z.R. Supervision: Z.R.

## Conflicts of Interest

The authors declare no conflicts of interest.

## Supporting information


**Data S1.** Supplementary Figures

**Table S1:** Predictions of Rat clock 1 and 2.

**Table S2:** Predictions of the intersection clock.

**Table S3:** Predictions of the rat rDNA clock.

**Table S4:** Weights of Rat clock 1.

**Table S5:** Weights of Rat clock 2.

**Table S6:** Promoter methylation data.

**Table S7:** Methylation percentages of the rat rDNA for the Levine et al. dataset.

**Table S8:** Methylation percentages of the rat rDNA for our LCR/HCR dataset.

**Table S9:** Genes associated with the Rat clock 1 CpGs sites.

**Table S10:** Genes associated with the Rat clock 2 CpGs sites.

## Data Availability

Raw and processed methylation data are available in the GEO database upon publication. Source data of the figures are available in the supplementary tables: Table S1 (predictions of Rat clocks 1 and 2), Table S2 (predictions of the intersection clock), Table S3 (predictions of the rat rDNA clock), Table S4 (weights of Rat clock 1), Table S5 (weights of Rat clock 2), Table S6 (promoter methylation data), Table S7 (methylation percentages of the rat rDNA for Levine et al.'s dataset), Table S8 (methylation percentages of the rat rDNA for our LCR/HCR dataset, Table S9 (genes associated with the Rat clock 1 CpGs sites), and Table S10 (genes associated with the Rat clock 2 CpGs sites). Any additional data are available from the corresponding author upon reasonable request.
