# Supplementary material for: Organ Specificity and Commonality of Epigenetic Aging in Low‐ and High‐Running Capacity Rats
Source: Aging Cell. 2025 Jun 8;24(8):e70110. doi: 10.1111/acel.70110 (PMC12341768; doi:10.1111/acel.70110)
Supplement: Supplementary file 1 — Data S1. Supplementary Figures [file ACEL-24-e70110-s002.docx]

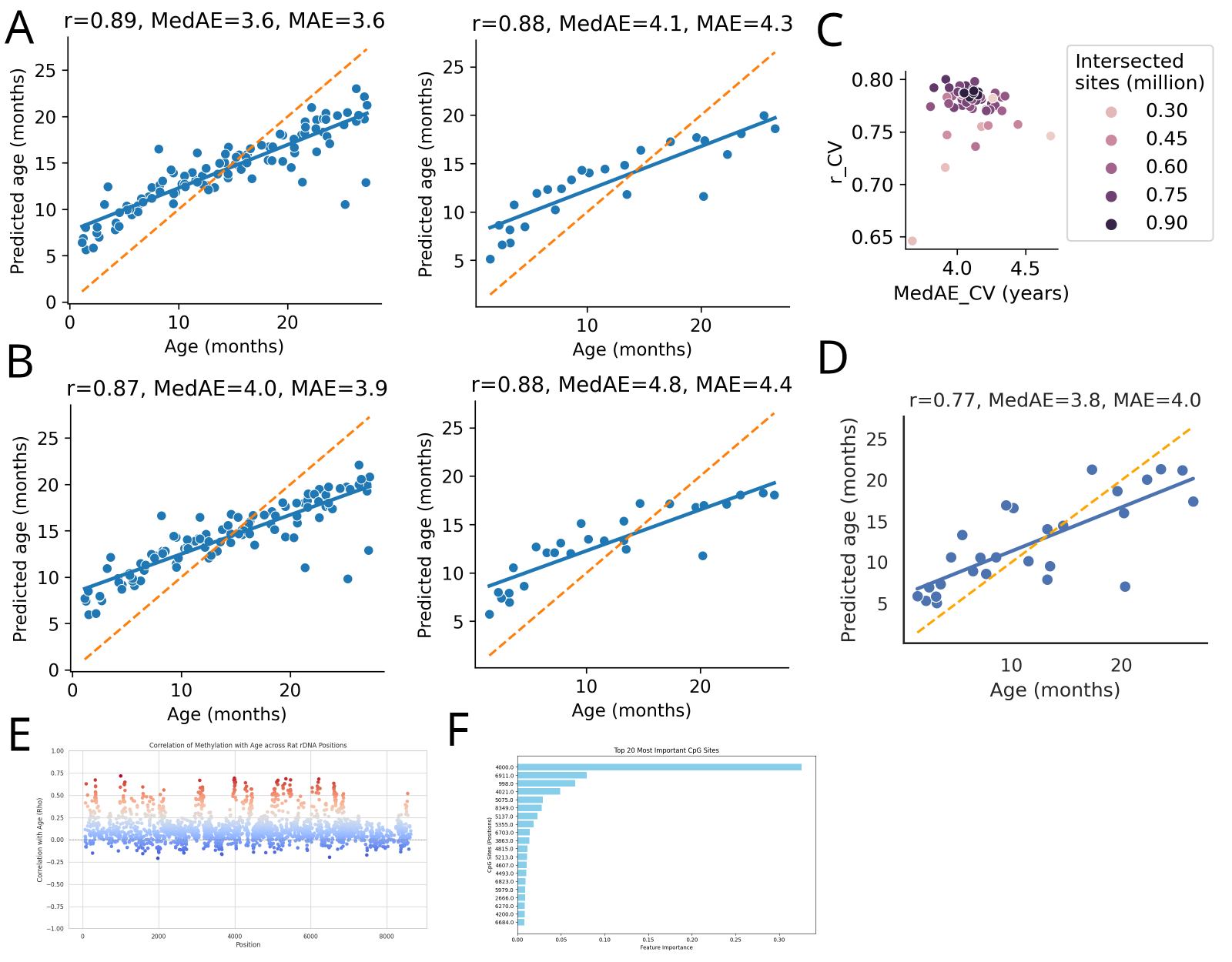


**Figure S1. Training and testing of the four clocks on the Levine et al. (2020) rat blood dataset**. **A.** Training and testing of the Rat clock 1. **B.** Training and testing of the Rat clock 2. **C.** Number of intersected CpG sites and cross-validation (CV) model performance of the intersection clock assessed by Pearson correlation coefficient (*r*) and median absolute error (MedAE). **D.** Predictions of the rat rDNA clock on the test set. **E.** Spearman correlation coefficent (Rho; y axis) between age and the methylation level of a CpG site located in a specific position (x-axis) within the rat rDNA sequence. **F.** The top 20 most important features (CpG site positions) of the final model (gradient boosting) of the rat rDNA clock.

**
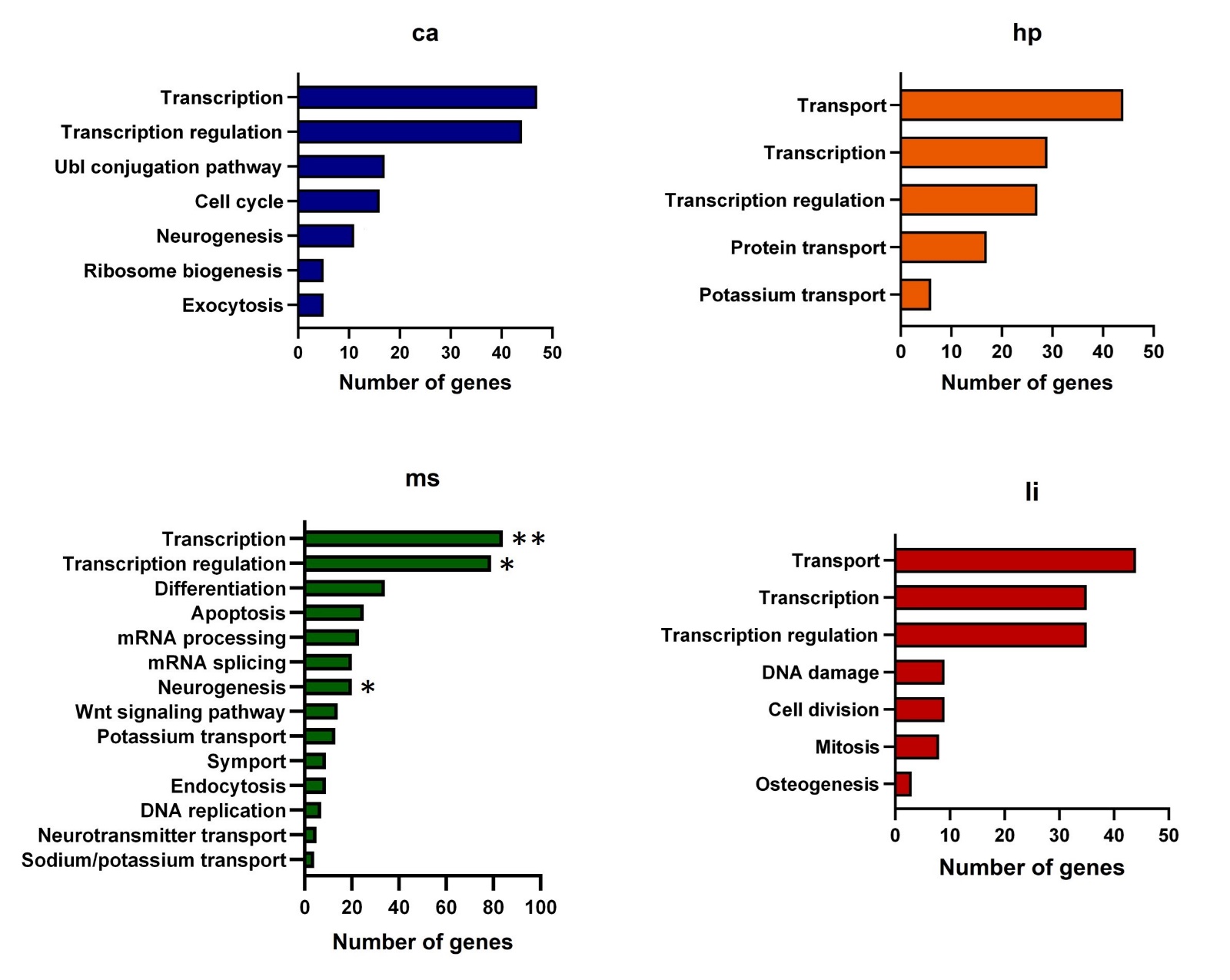
**

**Figure S2. Enrichment analysis of genes with significant promoter methylation differences between LCR and HCR rats across organs.** Gene sets were analyzed using the Database for Annotation, Visualization and Integrated Discovery (DAVID). Asterisks indicated significantly enriched by Benjamini-Hochberg corrected. Abbreviations: ca: heart, hp: hippocampus, ms: soleus muscle, li: large intestine. *: 0.01 < *p* ≤ 0.05, **: 0.001 < *p* ≤ 0.01.

**Figure S3. Inconsistency of epigenetic age acceleration across org
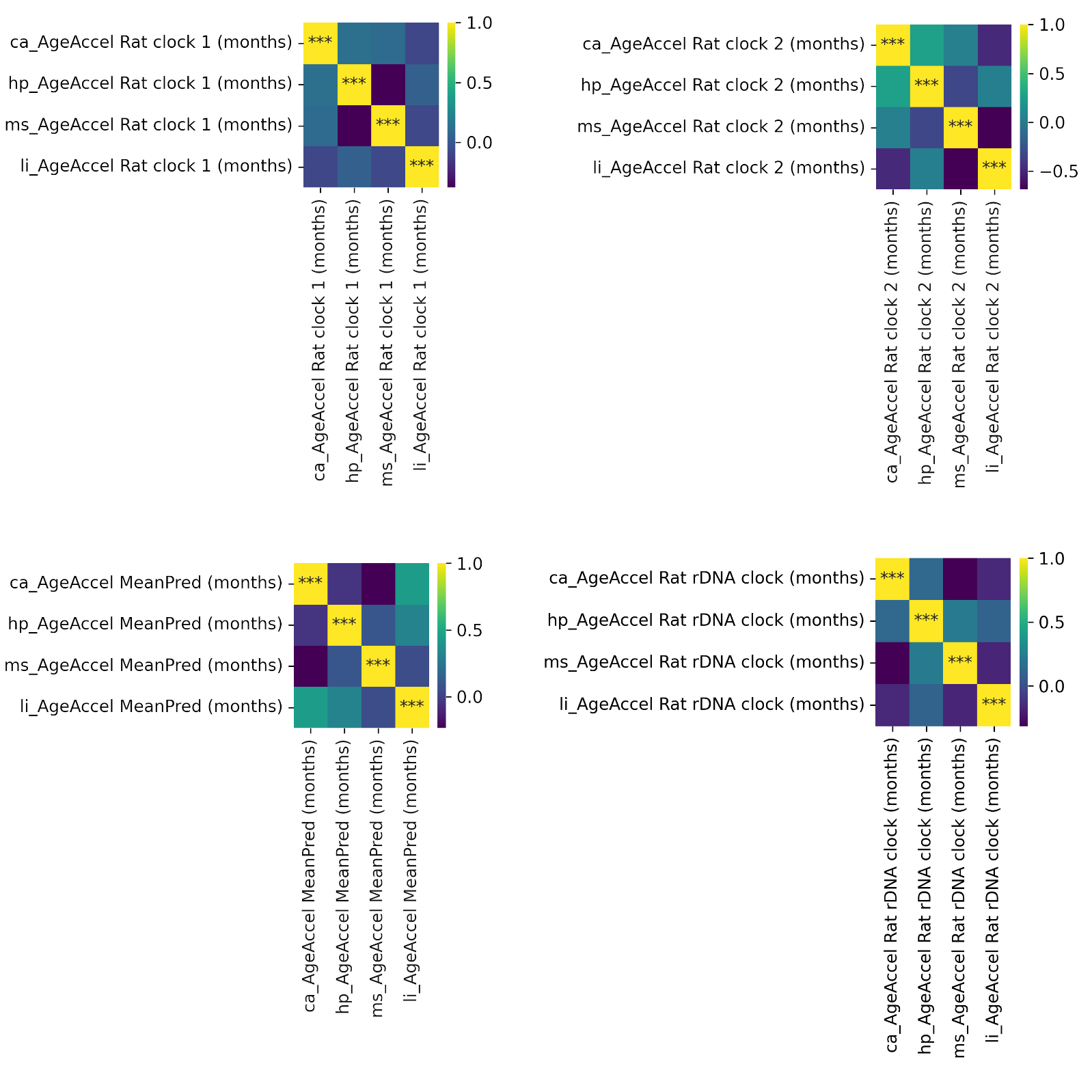
ans in the same individual.** We calculated the Pearson correlation coefficient between age accelerations of organ-pairs. The three plots are linked to the epigenetic age acceleration based on the four rat clocks (Rat clock 1, Rat clock 2, intersection clock, and rat rDNA clock, respectively). Abbreviations: ca: heart, hp: hippocampus, ms: soleus muscle, li: large intestine. *: 0.01 < *p* ≤ 0.05, **: 0.001 < *p* ≤ 0.01, ***: *p* ≤ 0.001, ****: *p* ≤ 0.0001, ns: not significant.

**Figure S4. Gene expression analysis of the top differently methylated genes between LCR and HCR rats. A-D.** Relative expression levels of the top differently methylated genes between LCR and HCR rat samples from cardiac muscle, hippocampus, and soleus muscle evaluated using real-time PCR (qRT-PCR) by normalized to *beta-actin and HPRT-1*. Only the genes are displayed where detectable expression was observed. Statistical analyses were performed using the Mann–Whitney test (**p < 0.01). Data are presented as mean ± SEM.
